# A Review on the Role of AFAP1-AS1 in the Pathoetiology of Cancer

**DOI:** 10.3389/fonc.2021.777849

**Published:** 2021-11-29

**Authors:** Soudeh Ghafouri-Fard, Tayybeh Khoshbakht, Bashdar Mahmud Hussen, Mohammad Taheri, Majid Mokhtari

**Affiliations:** ^1^ Department of Medical Genetics, School of Medicine, Shahid Beheshti University of Medical Sciences, Tehran, Iran; ^2^ Phytochemistry Research Center, Shahid Beheshti University of Medical Sciences, Tehran, Iran; ^3^ Department of Pharmacognosy, College of Pharmacy, Hawler Medical University, Erbil, Iraq; ^4^ Urology and Nephrology Research Center, Shahid Beheshti University of Medical Sciences, Tehran, Iran; ^5^ Institute of Human Genetics, Jena University Hospital, Jena, Germany; ^6^ Skull Base Research Center, Loghman Hakim Hospital, Shahid Beheshti University of Medical Sciences, Tehran, Iran

**Keywords:** AFAP1-AS1, cancer, biomarker, expression, ncRNA

## Abstract

AFAP1-AS1 is a long non-coding RNA which partakes in the pathoetiology of several cancers. The sense protein coding gene from this locus partakes in the regulation of cytophagy, cell motility, invasive characteristics of cells and metastatic ability. In addition to acting in concert with AFAP1, AFAP1-AS1 can sequester a number of cancer-related miRNAs, thus affecting activity of signaling pathways involved in cancer progression. Most of animal studies have confirmed that AFAP1-AS1 silencing can reduce tumor volume and invasive behavior of tumor cells in the xenograft models. Moreover, statistical analyses in the human subjects have shown strong correlation between expression levels of this lncRNA and clinical outcomes. In the present work, we review the impact of AFAP1-AS1 in the carcinogenesis.

## Introduction

Actin filament-associated protein 1 antisense RNA 1 (AFAP1-AS1, NC_000004.12) is a long non-coding RNA (lncRNA) which contributes in the pathoetiology of several cancers (1). It is transcribed from *AFAP1* gene locus on 4p16.1. It has two alternatively spliced variants. Its second exon overlaps with exons 14-16 of *AFAP1* gene. The motor fiber-associated protein encoded by *AFAP1* has been shown to organize a platform for joining a number of tumor-related proteins such as SRC and protein kinase C (2). This platform can influence the organization and activity of actin filaments, therefore participating in cytophagy, cell motility, invasive characteristics of cells and metastatic ability (3). Both AFAP1 and FAP1-AS1 participate in the carcinogenesis through modulation of related signaling pathways. AFAP1 has acknowledged roles in the pathogenesis of a number of cancers, namely breast (4) and prostate cancer (5), yet its expression has been found to decreased in gastric cancer samples (6). AFAP1-AS1 is mainly regarded as an oncogenic lncRNA (1). However, the oncogenic effect of this lncRNA is not necessarily exerted through AFAP1-dependent routes. A number of deletion type copy-number variants (CNVs) have been identified in *AFAP1-AS1* coding gene through application of whole genome sequencing (7). AFAP1-AS1 has been shown to affect several aspects of carcinogenesis through modulation of expression of cancer-related miRNAs. Since it has been shown to be dysregulated in diverse types of cancer, this lncRNA is a putative marker for a wide variety of cancers. Functional impacts of AFAP1-AS1 in the carcinogenesis have been appraised through knock-down and over-expression studies in cell lines and animal models. Moreover, the impact of AFAP1-AS1 deregulation has been assessed in human samples. In the present review, we discuss the role of AFAP1-AS1 in the carcinogenesis based on the evidence from these three types of studies.

## Cell Line Studies

### Lung Cancer

**Figure 1:**
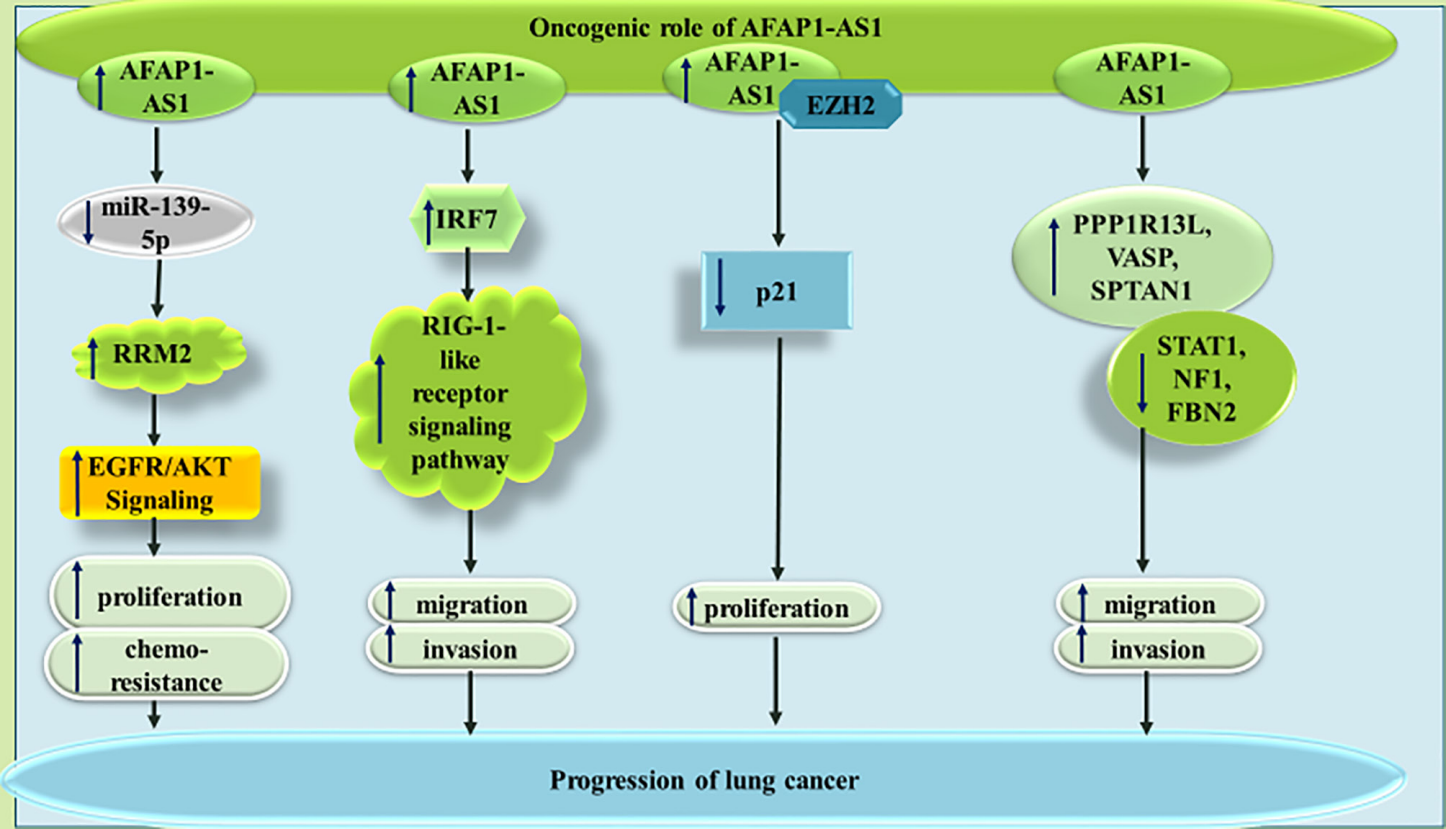
The oncogenic role of AFAP1-AS1 in lung cancer through modulation of expressions of RRM2, IRF7, p21 and PPP1R13L. The effects of AFAP1-AS1 on RRM2 expression is mediated through sponging miR-139-5p. This mode of action results in enhancement of cell proliferation, migration and invasiveness.

AFAP1-AS1 has been found to be over-expressed in non-small cell lung cancer (NSCLC) cells H1975, PC-9, A549, and SPCA-1 compared with the human non-tumorigenic lung epithelial cell line BEAS-2B. Functional studies in these cells have confirmed the ability of this lncRNA in binding with and sequestering miR-139-5p, a down-regulated miRNA in NSCLC samples. AFAP1-AS1 silencing and miR-139-5p up-regulation could similarly inhibit proliferation, colony forming ability and chemoresistance of NSCLC cells, while increasing their apoptosis. The sequestering impact of AFAP1-AS1 on miR-139-5p leads to up-regulation of RRM2, a protein which has been demonstrated to increase chemoresistance of NSCLC cells *via* activation of EGFR/AKT pathway (8). Another study in NSCLC has shown up-regulation of FAP1-AS1 parallel with down-regulation of IL-12 and up-regulation of IL-10 and IFN-γ. Functionally, AFAP1-AS1 has been shown to induce activity of IRF7, RIG-I-like receptor signals and Bcl-2. Cumulatively, AFAP1-AS1 enhances migration and invasive properties of NSCLC cells through activating IRF7 and the RIG-I-like receptor signaling pathway (9). Moreover, the interaction between AFAP1-AS1 and EZH2 and subsequent recruitment of EZH2 to the promoter of p21 has been shown to repress expression of p21 in this type of cancer (10). AFAP1-AS1 has also been shown to enhance expression of AFAP1 in lung cancer cells. Expression of AFAP1-AS1 in lung cancer cells is regulated through CpG methylation marks in its promoter, since the DNA methyltransferase inhibitor agent decitabine has been demonstrated to activate AFAP1-AS1 expression. AFAP1-AS1 has been reported to increase expression levels of pro-invasive genes PPP1R13L, VASP and SPTAN1, while decreasing expression levels of a number of anti-metastatic genes such as STAT1, NF1, and FBN2 (11). **Figure 1** summarizes the mentioned routes of participation of AFAP1-As1 in the pathogenesis of lung cancer.

**Figure 2:**
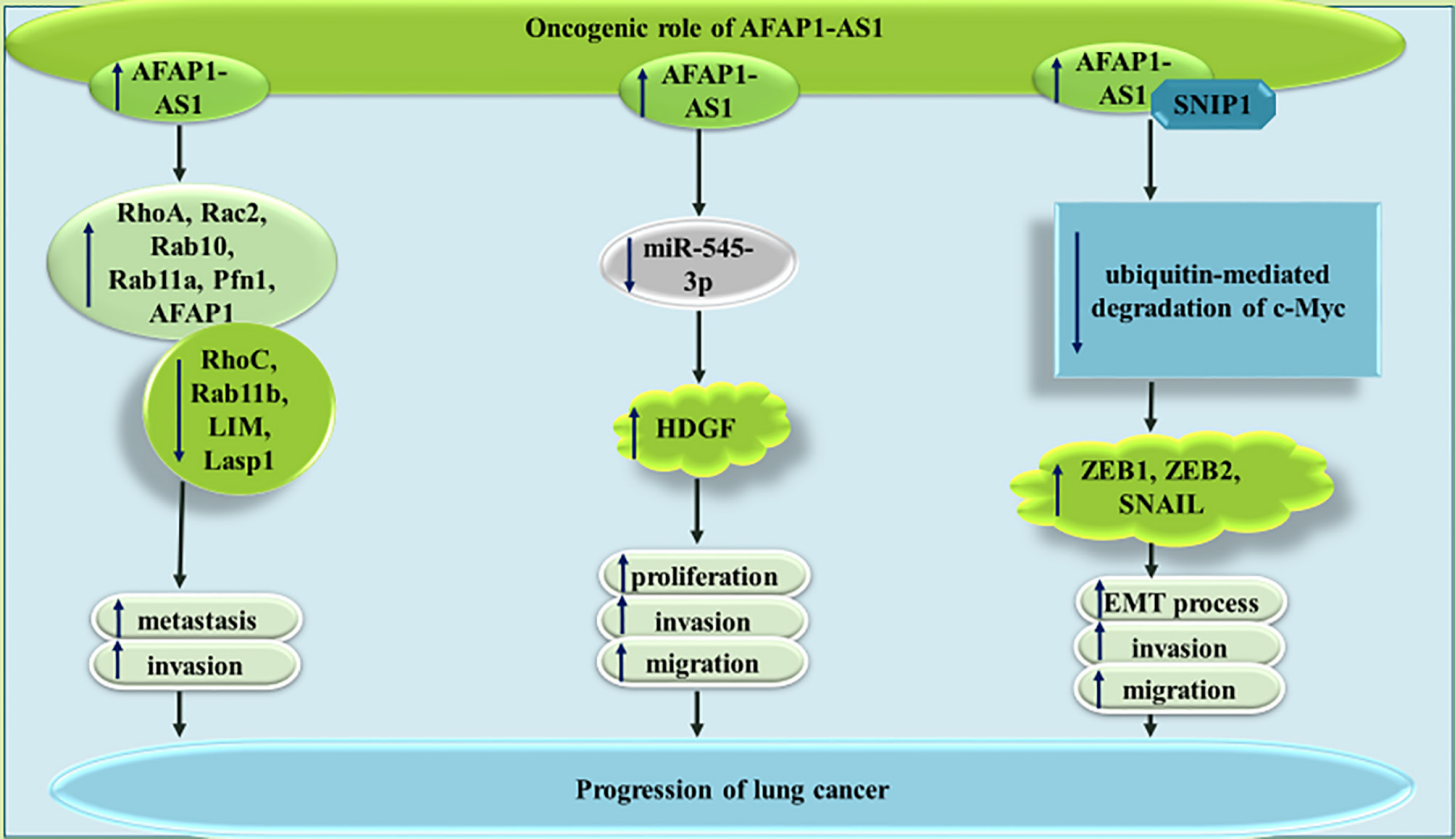
The oncogenic role of AFAP1-AS1 in lung cancer metastasis.

AFAP1-AS1 can also affect lung cancer through a variety of other mechanisms being summarized in **Figure 2**. For instance, AFAP1-AS1 has been shown to regulate expression of numerous members of the small GTPase proteins as well as those participating in the actin cytokeratin signaling. Thus, the promoting effect of AFAP1-AS1 on cancer metastasis is most probably exerted through modulation of actin filament integrity (12). GTPases harmonize several cellular processes, such as cell polarity, migration, and cell cycle transition, thus they can participate in the pathogenies of cancer (13). Moreover, cytokeratins as members of intermediate filament protein family have been shown to affect carcinogenesis. They can also been used as cancer biomarkers (14).

AFAP1-AS1 can also enhance expression of HDGF through decreasing miR-545-3p levels in lung cancer cells. Thus, AFAP1-AS1 silencing could inhibit progression of lung cancer through influencing activity of miR-545-3p/HDGF axis (15). Finally, AFAP1-AS1 can interact with Smad nuclear interacting protein 1 (SNIP1), a protein which suppresses ubiquitination and subsequent destruction of c-Myc. This function of AFAP1-AS1 leads to over-expression of c-Myc, increase in ZEB1, ZEB2, and SNAIL levels, and enhancement of epithelial to mesenchymal transition (EMT) (16).

### Breast Cancer

**Figure 3:**
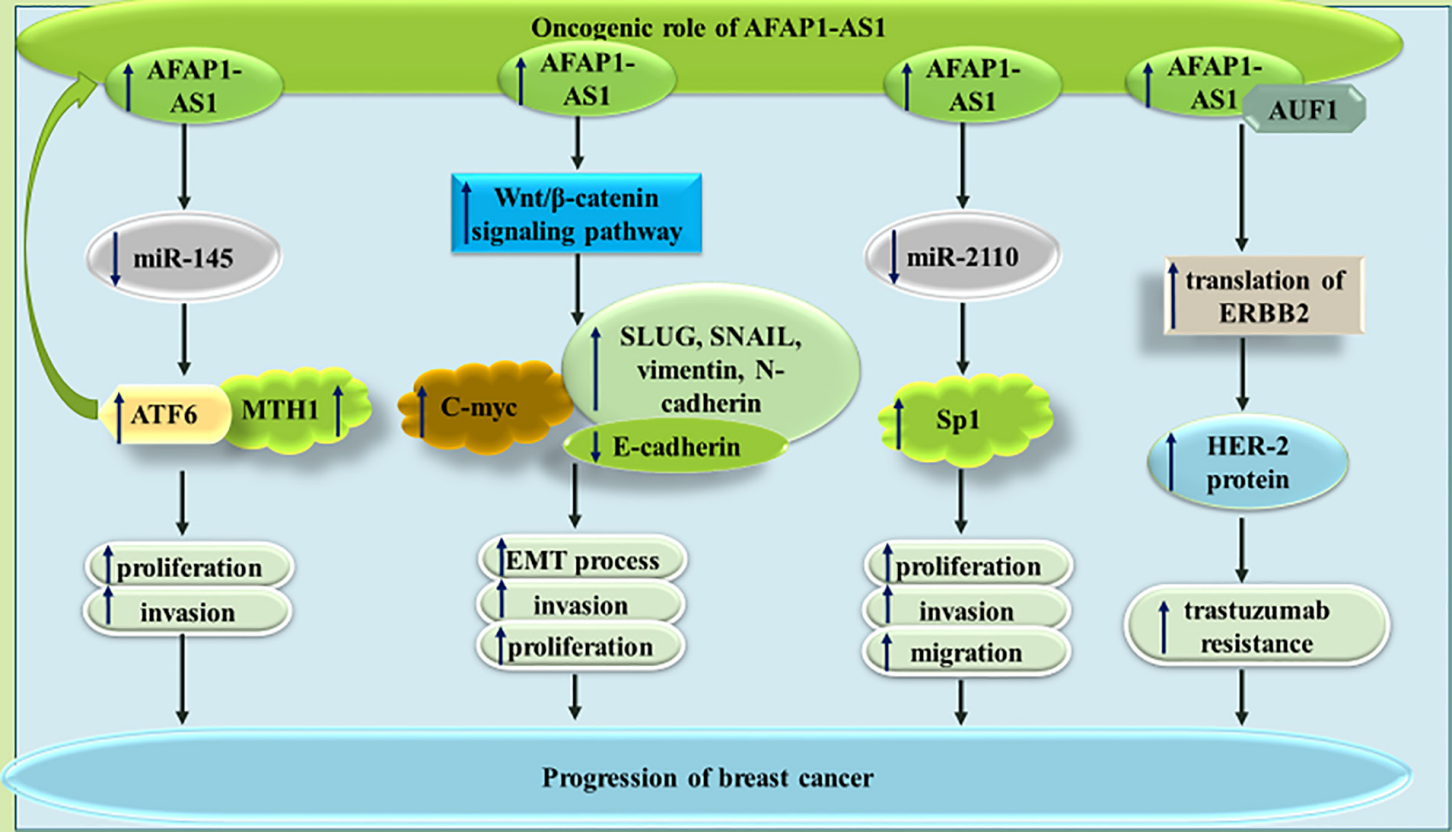
The impact of AFAP1-AS1 in breast cancer progression and resistance to therapy. In addition to increasing cell proliferation and invasion, this lncRNA can increase expression of Her-2 protein, thus increasing resistance to trastuzumab.

In breast cancer cells, AFAP1-AS1 silencing could decrease proliferation and migratory potential, and increase cell apoptosis. miR-497-5p has been recognized as a target of AFAP1-AS1 in breast cancer cells. Since this miRNA targets SEPT2, AFAP1-AS1 up-regulation results in up-regulation of SEPT2 (17). miR-145 is another target of AFAP1-AS1 in triple negative breast cancer cells (TNBC) MDA-MB-231 breast cancer cells. According to the results of luciferase reporter assay, miR-145 can directly target MTH1. Thus, the effects of AFAP1-AS1 in enhancement of proliferation and invasiveness of TNBC are exerted through miR-145/MTH1 axis (18). Moreover, in this type of cancer, AFAP1-AS1 can sequester miR-2110 to enhance expression of Sp1 (19). AFAP1-AS1 has also been shown to enhance EMT of TNBC cells *via* influencing Wnt/β-catenin signaling (20). Finally, AFAP1-AS1 has been found to have significant over-expression in trastuzumab-resistant breast cancer cells versus responsive cells. Expression of this lncRNA has been enhanced by H3K27ac at its promoter. Most notably, trastuzumab resistant cells have been shown to secrete AFAP1-AS1 into exosomes, thus disseminating trastuzumab resistance in other cells. The impact of exosomal AFAP1-AS1 in induction of trastuzumab resistance is exerted *via* its interaction with AUF1 and subsequent induction of ERBB2 translation (21). **Figure 3** depicts the impact of AFAP1-AS1 in carcinogenesis and therapy resistance of breast cancer cells.

### Osteosarcoma

**Figure 4:**
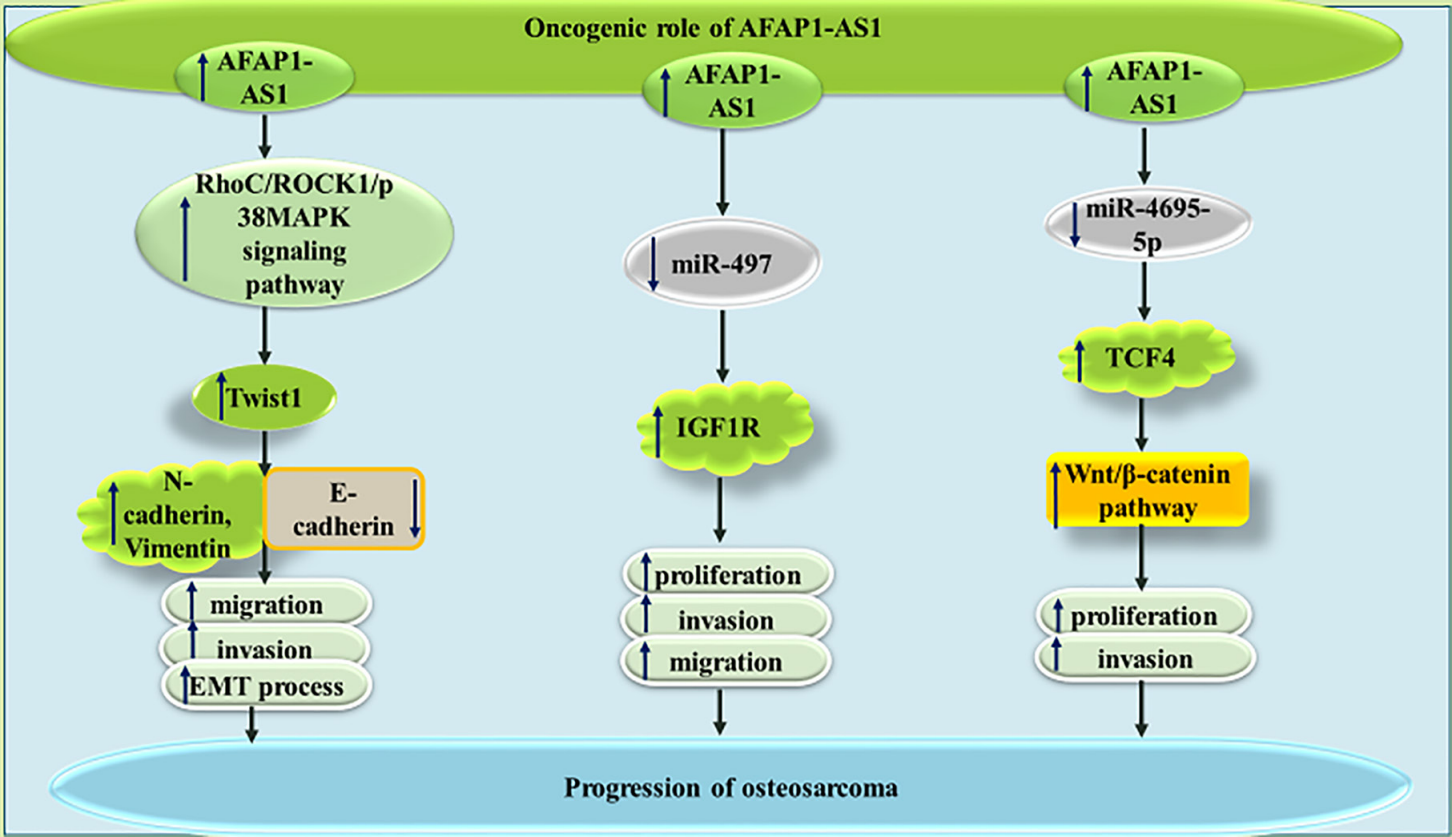
The oncogenic role of AFAP1-AS1 in osteosarcoma is exerted through modulation of RhoC/ROCK1/p38MAPK/Twist1 cascade as well as sponging miR-497 and miR-4695-5p.

In MNNG/HOS and U2OS osteosarcoma cells, AFAP1-AS1 has been found to promote tumorigenesis *via* influencing RhoC/ROCK1/p38MAPK/Twist1 cascade (22). The AFAP1-AS1-mediated increase in Twist1 can enhance expression of N-cadherin and Vimentin, while diminishing E-cadherin levels, thus promoting EMT of osteosarcoma cells (22). Moreover, AFAP1-AS1 can sequester miR-497 and miR-4695-5p in these cells, therefore increasing expressions of IGF1R and TCF4, respectively (23, 24). The latter can activate Wnt-β catenin pathway and increase both proliferation and invasive abilities of osteosarcoma cells (24). **Figure 4** depicts the oncogenic role of AFAP1-AS1 in osteosarcoma.

### Gastric Cancer

**Figure 5:**
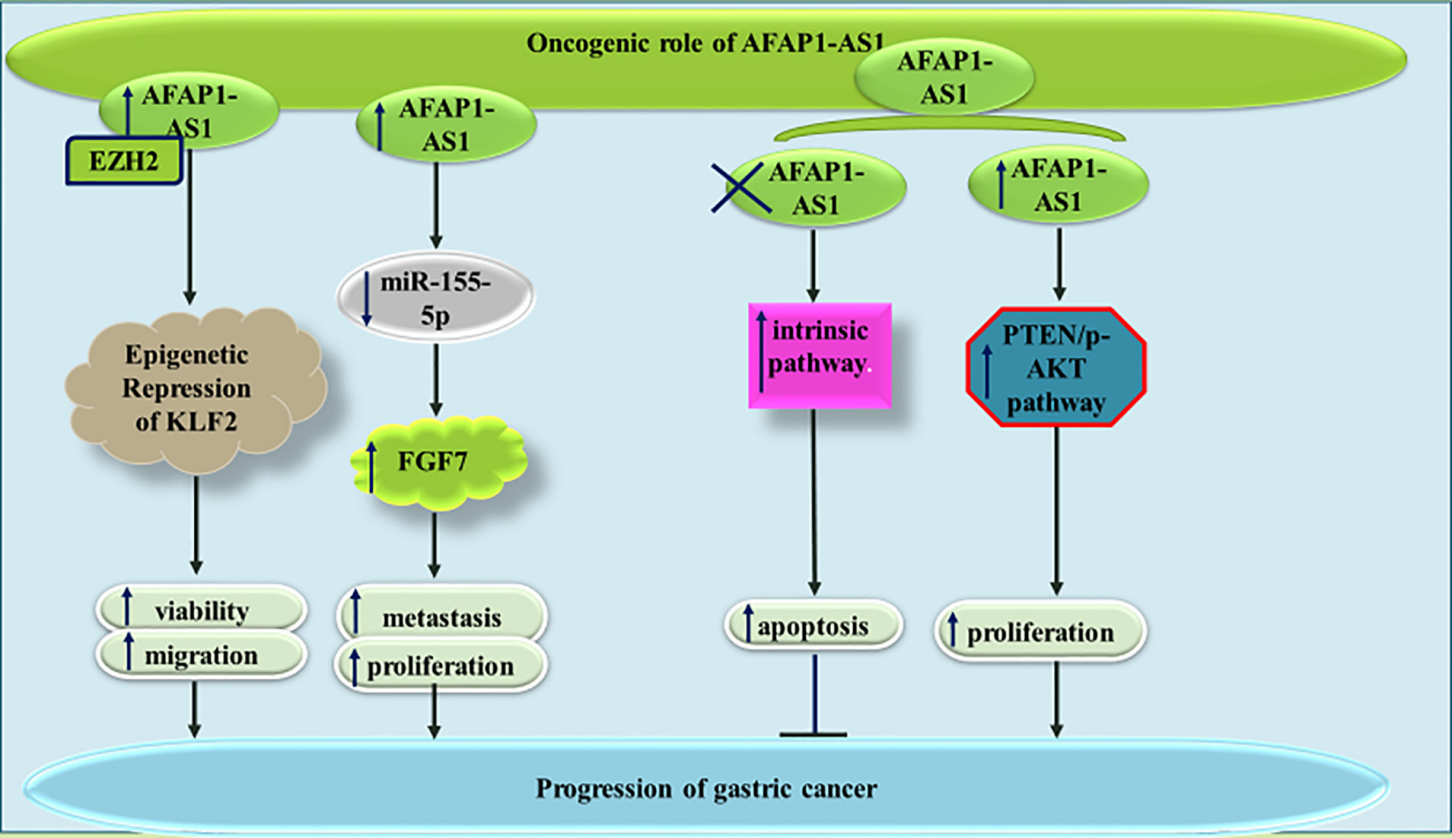
The oncogenic role of AFAP1-AS1 in gastric cancer is exerted through repression of KLF2, sponging miR-155-5p and enhancing activity of PTEN/p-AKT pathway.

Similarly, AFAP1-AS1 has an oncogenic role in gastric cancer. AFAP1-AS1 silencing has significantly suppressed proliferation and cell cycle transition in this kind of cancer. Besides, reduction in the levels of this lncRNA can inhibit invasive capacity through affecting EMT (25). Down-regulation of KLF2 is another mechanism by which AFAP1-AS1 enhances proliferative and migratory aptitudes of gastric cancer cells (26). AFAP1-AS1 silencing in gastric cancer cells has led to a significant increase in the levels of Bax, cleaved PARP, Caspase 3, and Caspase 9, while decreasing Bcl-2 level. AFAP1-AS1 silencing has also reduced p-AKT levels and enhanced expression of PTEN in gastric cancer cells. Taken together, AFAP1-AS1 regulates proliferation and apoptotic processes in gastric cancer cell through PTEN/p-AKT cascade (27). AFAP1-AS1 can also promote proliferation and metastatic ability of gastric cancer cell through sequestering miR-155-5p and enhancing expression of FGF7 (28). **Figure 5** shows the oncogenic role of AFAP1-AS1 in gastric cancer.

### Esophageal Cancer

AFAP1-AS1 have also been shown to bind with miR-26a, therefore influencing expression of its target gene, i.e. ATF2. Exosomes originated from M2 macrophages have higher expression of AFAP1-AS1 and ATF2 and reduced expression of miR-26a, compared with M1 macrophages. These exosomes could transfer AFAP1-AS1 to esophageal cancer cells, thus downregulating miR-26a and enhancing ATF2 levels in the recipeint cells. These expression changes affect phenotype of esophageal cancer cells (29). The regulatory role of AFAP1-AS1 on miR-498/VEGFA axis is another mechanism of participation of this lncRNA in the pathetiology of esophageal cancer (30).

### Other Types of Cancers

In prostate cancer cells, AFAP1-AS1 has been shown to promote sequester miR-195-5p (31) and miR-512-3p (32), thus affecting malignnat behavious of these cells.

**Figure 6:**
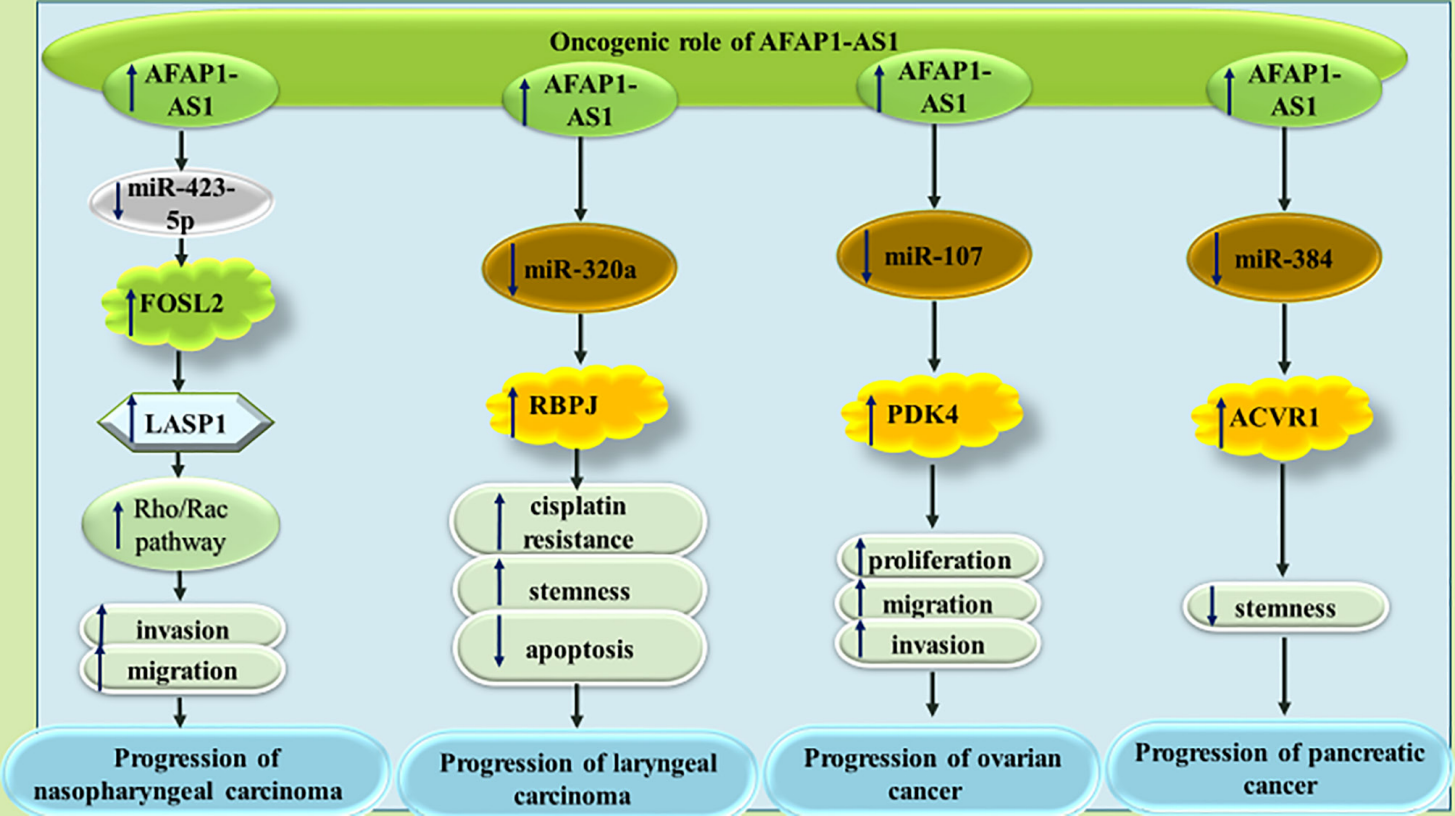
The oncogenic role of AFAP1-AS1 in nasopharyngeal carcinoma, laryngeal carcinoma, ovarian cancer and pancreatic cancer. In all types of mentioned cancers, AFAP1-AS1 can act as molecular sponge for tumor suppressor miRNAs.

A number of other miRNAs, namely miR-423-5p (33), miR-320a (34), miR-107 (35) and miR-384 (36) have been found to be sequestered by AFAP1-AS1 in different cancer tissues (**Figure 6**).

**Table 1** summarizes the results of studies which appraised oncogenic roles of AFAP1-AS1 in different tissues.

**Table 1:** Outlines of papers which judged expression of AFAP1-AS1 in cell lines.

Tumor type	Interactions	Cell lines	Effects	Reference
Non-small Cell Lung Cancer	miR-139-5p, RRM2, EGFR/AKT signaling pathway	H1975, PC-9, A549, SPCA-1, BEAS-2B	Δ AFAP1-AS1: ↓ proliferation, ↓ chemo-resistance, ↑ apoptosis	(8)
	_	A549, H1975, H1650, H1395, H12994	Δ AFAP1-AS1: ↓ proliferation ↑ AFAP1-AS1: ↑ invasion, ↑ migration, ↓ apoptosis	(9)
	p21, EZH2	16HBE, A549, SPC-A, H1299	Δ AFAP1-AS1: ↓ proliferation, ↑ cell cycle arrest	(10)
	PPP1R13L, VASP, SPTAN1, STAT1, NF1, FBN2, AFAP1	H1299, PC9, H1975, 293T	Δ AFAP1-AS1: ↓ invasion, ↓ migration ↑ AFAP1-AS1: ↑ invasion, ↑ migration	(11)
	HBP1	16HBE, A549, SPC‐A1, PC‐9, H1299, H1975	Δ AFAP1-AS1: ↓ proliferation, ↓ migration, ↑ G0/G1 cell cycle arrest, ↑ apoptosis	(37)
Lung cancer	AFAP1, KRT1	A549, H1299 and H460, 95-D, 16HBE	Δ AFAP1-AS1: ↓ proliferation, ↓ migration	(38)
	RhoA, Rac2, Rab10, Rab11a, Rhogdi proteins, Pfn1, RhoC, Rab11b, LIM, Lasp1	A549	Δ AFAP1-AS1: ↓ invasion, ↓ migration, ↓ metastasis	(12)
	miR-545-3p, HDGF	_	Δ AFAP1-AS1: ↓ proliferation ↓ invasion, ↓ migration, ↑ apoptosis	(15)
	SNIP1, c-Myc, ZEB1, ZEB2, SNAIL	A549, PC9	Δ AFAP1-AS1: ↓ invasion, ↓ migration, ↓ EMT process	(16)
	_	H1915, HCC827	Δ AFAP1-AS1: ↓ invasion, ↓ growth, ↑ apoptosis	(39)
Breast cancer (BC)	_	MCF-10A, MCF-7, SK-RB-3, MDA-MB231, MDA-MB-468	Δ AFAP1-AS1: ↓ proliferation, ↓ colony formation, ↓ metastasis ↑ apoptosis, did not affect AFAP1 expression, did not affect actin filament integrity	(40)
	miR-497-5p	HCC70, BT-549, MCF-7, MDA-MB-231, MCF-10A	Δ AFAP1-AS1: ↓ proliferation, ↓ migration, ↑ apoptosis	(17)
	miR-145, MTH1, ATF6	MDA-MB-231, MDA-MB-468, MDA-MB-435S, and HCC1937, MCF-10A	Δ AFAP1-AS1: ↓ viability, ↓ colony formation, ↓ invasion	(18)
	Wnt/β-catenin signaling pathway, C-myc, SLUG, SNAIL, vimentin, fibronectin, N-cadherin, E-cadherin	184A1, MCF-10A, BT474, MCF-7, T47D, BT483, BT20, MDA-MB-468, BT549, MDA-MB-231	Δ AFAP1-AS1: ↓ proliferation ↓ invasion, ↓ migration, ↓ EMT process, ↑ apoptosis	(20)
	miR-2110, Sp1	MCF-10A, BT-549, MDA–MB-468	Δ AFAP1-AS1: ↓ proliferation ↓ invasion, ↓ migration	(19)
	ERBB2, AUF1	KBR-3, BT474,	Δ AFAP1-AS1: ↓ trastuzumab resistance	(21)
Osteosarcoma	Twist1, N-cadherin and Vimentin, E-cadherin, RhoC/ROCK1/p38MAPK signaling pathway	MNNG/HOS, MG63, SaOS-2, hFOB 1.19	Δ AFAP1-AS1: ↓ proliferation, ↓ invasion, ↓ migration, ↓ actin filament integrity, ↓ EMT process, ↓ VM formation capacity, ↑ apoptosis, ↑ G0/G1 cycle arrest	(22)
	miR-497, IGF1R	MG-63, 143B, U2OS, Saos-2, hFOB 1.19	Δ AFAP1-AS1: ↓ proliferation ↓ invasion, ↓ migration, ↑ apoptosis,	(23)
	miR-4695-5p, TCF4, Wnt/β-catenin pathway	hFOB 1.19, Saos-2, U2OS, MG-63, 143B	Δ AFAP1-AS1: ↓ proliferation ↓ invasion	(24)
Esophageal cancer (EC)	miR-26a, ATF2	PBMCs, KYSE410	Δ AFAP1-AS1 in M2 Macrophage-Derived Exosomes: ↓ invasion, ↓ migration, ↓ metastasis	(29)
	miR-498, VEGFA	HET-1A, Eca109, KYSE-30	Δ AFAP1-AS1: ↓ proliferation, ↓ Migration, ↑ apoptosis	(30)
	_	ECA‐109, TE‐1, HEEC	Δ AFAP1-AS1: ↓ proliferation, ↑ apoptosis	(41)
	_	OE-33, SK-GT-4, FLO-1, HEEpic	Δ AFAP1-AS1: ↓ proliferation, ↓ invasion, ↓ anchorage-dependent growth did not affect the expression level of AFAP1	(42)
Gastric cancer (GC)	KLF2, EZH2	GES-1, AGS and SGC-7901	Δ AFAP1-AS1: ↓ proliferation, ↓ invasion, ↓ viability, ↑ apoptosis	(26)
	intrinsic pathway, PTEN/p-AKT Pathway	AGS, MGC-803, SGC-7901, BGC-823, GES-1	Δ AFAP1-AS1: ↓ proliferation, ↑ apoptosis	(27)
	_	MKN-45, MGC-803 and AGS	Δ AFAP1-AS1: ↓ proliferation, ↓ migration, ↓ invasion, ↑ G0/G1 phase arrest, ↑ apoptosis	(43)
	_	AGS, BGC823, MGC-803, SGC-7901, GES-1	Δ AFAP1-AS1: ↓ proliferation, ↓ invasion, ↓ EMT process, ↓ cell cycle progress	(25)
	miR-155-5p, FGF7	MKN-28, BGC-823, MGC-803, SGC-7901, GES-1	Δ AFAP1-AS1: ↓ proliferation, ↓ migration, ↓ invasion	(28)
	_	GES-1, HGC-27, MGC-803, BGC-823, SGC-7901	Δ AFAP1-AS1: ↓ proliferation, ↓ migration, ↓ invasion	(44)
	_	GES-1, AGS, BGC-823, MKN-45, SGC-7901	Δ AFAP1-AS1: ↓ proliferation, ↓ migration, ↓ invasion, ↓ EMT process	(45)
Prostate cancer	miR-195-5p, FKBP1A	PC3, DU145	Δ AFAP1-AS1: ↑ PTX sensitivity, ↑ apoptosis, ↓ migration, ↓ invasion	(31)
	miR-512-3p	22RV1	Δ AFAP1-AS1: ↓ proliferation, ↓ migration, ↓ invasion, ↑ G0/G1 phase arrest	(32)
Nasopharyngeal carcinoma (NPC)	YAP, KAT2B, RBM3	HNE-1, C666-1, SUNE-1, CNE-1, CNE-2, NP69	Δ AFAP1-AS1: ↓ proliferation	(46)
	miR-423-5p, Rho/Rac signaling, FOSL2, LASP1	5-8F, HNE2	↑ AFAP1-AS1: ↑ migration, ↑ invasion	(33)
	AFAP1, RhoA, Rac2, Rab10, Rab11a, Rhogdi, Pfn1, RhoC, Rab11b, Lasp1	5-8F, HNE2 and HK-1	Δ AFAP1-AS1: ↓ migration, ↓ invasion, ↓ stress filament integrity	(47)
Endometrial carcinoma (EC)	miR-545-3p, VEGFA	Ishikawa, HEC-1-B, HEC1-A, AN3-CA, hEEC,	Δ AFAP1-AS1: ↓ proliferation, ↓ migration, ↓ invasion, ↓ angiogenesis	(48)
Cholangiocarcinoma (CCA)	AFAP1	HuCCT1, TFK-1, HIBEpic	Δ AFAP1-AS1: ↓ proliferation, ↓ migration, ↓ invasion, ↓ stress filament integrity	(49)
	MMP-2, MMP-9	QBC939, CCLP1, HuCC-T1 and RBE, BEC, 293T	Δ AFAP1-AS1: ↓ proliferation, ↓ migration, ↓ invasion, ↑ G0/G1 phase arrest	(50)
Colorectal cancer (CRC)	GAS8-AS1	CR4 (Sigma-Aldrich, USA), RKO (ATCC, USA)	↑ AFAP1-AS1: ↑ proliferation	(51)
	_	HCT116, SW480	Δ AFAP1-AS1: ↓ proliferation, ↑ G0/G1 phase arrest	(52)
	AFAP1	HCT116, SW480	Δ AFAP1-AS1: ↓ proliferation, ↓ migration, ↓ invasion	(53)
	EZH2	LOVO, SW1116, SW480, HCT116, SW620, HT29	Δ AFAP1-AS1: ↓ proliferation, ↑ cell-cycle arrest	(54)
Colon cancer	actin-cytokeratin signaling pathway, E-cadherin, vimentin, MMP9, ZEB1, ZO-1, β-catenin	SW480, SW620, HCT116, HT-29	Δ AFAP1-AS1: ↓ proliferation, ↓ migration, ↓ invasion	(55)
Hepatocellular carcinoma (HCC)	N-cadherin, vimentin, E-cadherin, CRKL, Ras, MEK, c-Jun	Huh7, HepG2, HCCLM3, LO2	Δ AFAP1-AS1: ↓ proliferation, ↓ migration, ↓ invasion, ↓ EMT process	(56)
	RhoA/Rac2 signaling	SMCC7721 and HepG2	Δ AFAP1-AS1: ↓ proliferation, ↓ invasion, ↑ S phase arrest, ↑ apoptosis	(57)
	_	LO2, SMMC-7721, Bel-7402, MHCC-97 L, MHCC-97H	Δ AFAP1-AS1: ↓ proliferation, ↓ migration, ↓ invasion	(58)
Cervical cancer (CC)	RhoA/Rac2 signaling, Vimentin, β-catenin, ZO-1	ATCC no. CCL-2,	Δ AFAP1-AS1: ↓ migration, ↓ invasion, ↓ EMT process	(59)
Laryngeal carcinoma	miR‐320a, RBPJ	HEp‐2	Δ AFAP1-AS1: ↓ stemness, ↓ cisplatin resistance, ↑ apoptosis	(34)
Thyroid cancer	_	K-1, TPC-1, SW579, FTC133, XTC-1, l Nthy-ori3-1	Δ AFAP1-AS1: ↓ proliferation, ↓ migration, ↓ EMT process, ↑ apoptosis	(60)
Glioma	_	U87MG, U251, SHG-44, A172	Δ AFAP1-AS1: ↓ invasion	(61)
Ovarian cancer (OC)	_	SKOV3, OV90, TOV112D, ES2	Δ AFAP1-AS1: ↓ proliferation, ↑ apoptosis ↑ AFAP1-AS1: ↑ proliferation	(62)
	miR-107, PDK4	IOSE80, COV504, OVISE, OV90 and SKOV3	Δ AFAP1-AS1: ↓ proliferation, ↓ migration, ↓ invasion	(35)
Pancreatic cancer (PC)	miR-384, ACVR1	SW1990, Capan-1, AsPC-1, MIAPaCa-2, PANC-1, HPC-Y5	Δ AFAP1-AS1: ↓ stemness	(36)
	ZEB1, N-cadherin, E-cadherin, MMP-2, MMP-9, Slug, Snail	BxPC-3, PANC-1	Oridonin-induced Δ AFAP1-AS1: ↓ proliferation, ↓ migration, ↓ EMT process, ↑ apoptosis, ↑ cell cycle arrest	(63)
	miR-133a, IGF1R	AsPC-1, BxPC-3, PANC-1, PaCa-2 and SW1990	Δ AFAP1-AS1: ↓ proliferation, ↓ invasion, ↓ metastasis, ↑ apoptosis	(64)
	EGFR/Akt signaling, miR‐146b‐5p	ASPC‐1, BxPC‐3, HPAC, MiaPaCa‐2, HPDE6‐C7	CUB-induced Δ AFAP1-AS1: ↓ proliferation, ↑ cell cycle arrest	(65)
Pancreatic ductal adenocarcinoma (PDAC)	_	Panc1, MIAPaCa-2, Capan2, SW1990, BXPC-3, HPDE6	Δ AFAP1-AS1: ↓ proliferation, ↓ migration, ↓ invasion	(66)
Renal cell carcinoma (RCC)	PTEN/AKT signaling	HK2, 786-O, Caki-1, ACHN, A498	Δ AFAP1-AS1: ↓ proliferation, ↓ migration, ↓ invasion, ↓ EMT process	(67)
Gallbladder cancer (GBC)	_	NOZ, H69, GBC-SD, SGC-996	Δ AFAP1-AS1: ↓ proliferation, ↓ invasion, ↓ epithelial phenotype to mesenchymal phenotype	(68)
Pituitary adenoma	miR-103a-3p, PI3K/AKT Signaling Pathway	GH3 and MMQ	Δ AFAP1-AS1 + miR-103a-3p inhibitor: ↑ proliferation, ↑ cell cycle progression, ↓ apoptosis	(69)
	PTEN/PI3K/AKT signaling pathway	GH3, MMQ	Δ AFAP1-AS1: ↓ proliferation, ↑ cell cycle arrest, ↑ apoptosis	(70)
Melanoma	miR-653-5p, RAI14, E-cadherin, N-cadherin, Ki67	HEMa-LP, A375, M21, B16F10, SK-MEL-2	Δ AFAP1-AS1: ↓ proliferation, ↓ migration, ↓ invasion	(71)
Retinoblastoma	_	Weri-Rb1 and Y79, ARPE-19, HRMECs	Δ AFAP1-AS1: ↓ proliferation, ↓ migration, ↓ invasion	(72)
Tongue squamous cell carcinoma (TSCC)	Wnt/β-catenin, SLUG, SNAIL1, VIM, CADN, ZEB1, ZEB2, and TWIST1	SCC-15, Tca8113, SCC-4, SCC-9, CAL-27	Δ AFAP1-AS1: ↓ proliferation, ↓ migration, ↓ invasion, ↑ G0/G1 cell cycle arrest	(73)
Oral squamous cell carcinoma (OSCC)	miR-145, HOXA1	SCC9, SCC15, SCC25, HOKs	Δ AFAP1-AS1: ↓ proliferation, ↓ migration, ↓ invasion	(74)

(Δ: knock-down, CuB: Cucurbitacin B).

## Animal Studies

Investigations, particularly those conducted in BALB/c nude mice models have verified the oncogenic roles of AFAP1-AS1 in different types of cancers. AFAP1-AS1 knock-down has consistently led to significant reduction in tumor size/weight, attenuation of tumor growth rate and enhancement of response of cancer cells to therapeutic modalities (**Table 2**). In NSCLC, AFAP1-As1 silencing not only reduces tumorigenicity, but also confers chemosensitivity (8). Moreover, its silencing can affect IRF7 and RIG-I-like receptor signals (9). In breast cancer, AFAP1-AS1 down-regulation can affect trastuzumab resistance (21).

**Table 2:** Outlines of studies which tested function of AFAP1-AS1 in xenografts.

Tumor Type	Animal models	Results	Reference
Non-small Cell Lung Cancer	male athymic nude BALB/c mice	Δ AFAP1-AS1: ↓ tumorigenicity, ↓ chemo-resistance	(8)
	_	Δ AFAP1-AS1: ↓ mRNA and protein of IRF7 and RIG-I-like receptor signals	(9)
		↑ AFAP1-AS1: ↑ mRNA and protein of IRF7 and RIG-I-like receptor signals	
	male BALB/c nude mice	Δ AFAP1-AS1: ↓ tumor volume, ↓ tumor weight	(10)
	BALB/c nude mice	Δ AFAP1-AS1: ↓ tumor weight, ↓ tumor size	(37)
Lung cancer	BALB/c nude mice	Δ AFAP1-AS1: ↓ tumor volume, ↓ tumor weight, ↓ tumor growth	(38)
	murine xenograft mice	Δ AFAP1-AS1: ↓ tumor growth	(15)
	female nude mice	Δ AFAP1-AS1: ↓ metastatic nodules	(16)
Breast cancer (BC)	female nude mice	Δ AFAP1-AS1: ↓ tumor growth	(17)
	Female BALB/c nude mice	Δ AFAP1-AS1: ↓ tumor growth	(18)
	female nude mice	Δ AFAP1-AS1: ↓ tumor growth, ↓ tumor weight	(20)
	BALB/C specific-pathogen-free nude mice	Δ AFAP1-AS1: ↓ tumor volume, ↓ tumor weight, ↓ tumor growth	(19)
	male BALB/c nude mice	Δ AFAP1-AS1: ↓ tumor resistance, ↓ metastasis	(21)
Osteosarcoma	female BALB/c nude mice	Δ AFAP1-AS1: ↓ tumor growth, ↓ invasion	(22)
	male athymic BALB/c nude mice	Δ AFAP1-AS1: ↓ tumor volume, ↓ tumor weight, ↓ tumor growth	(23)
	female BALB/c nude mice	Δ AFAP1-AS1: ↓ tumor size, ↓ tumor weight	(24)
Esophageal cancer (EC)	_	Δ AFAP1-AS1: ↓ATF2, ↑ miR-26a	(29)
Gastric cancer (GC)	male BALB/c nude mice	Δ AFAP1-AS1: ↓ tumor volume, ↓ tumor weight, ↓ tumor growth	(44)
Prostate cancer	nude mice	Δ AFAP1-AS1: ↓ tumor volume, ↓ tumor weight, ↑ C-caspase 3	(31)
Nasopharyngeal carcinoma (NPC)	male BALB/C nude mice	↑ AFAP1-AS1: ↑ metastasis	(33)
	nude mice	Δ AFAP1-AS1: ↓ number and size of the metastatic foci	(47)
Endometrial carcinoma (EC)	male BALB/c nude mice	Δ AFAP1-AS1: ↓ tumor volume, ↓ tumor weight	(48)
Cholangiocarcinoma (CCA)	female BALB/c/nu nude mice	Δ AFAP1-AS1: ↓ tumor volume, ↓ tumor weight, ↓ number and size of the metastatic foci	(49)
	female BALB/c athymic nude mice	Δ AFAP1-AS1: ↓ tumor volume, ↓ tumor weight	(50)
Colorectal cancer (CRC)	male C57BL/6 nude mice	Δ AFAP1-AS1: ↓ tumor volume, ↓ tumor weight	(53)
	female BALB/c-nude mice	Δ AFAP1-AS1: ↓ tumor growth	(54)
Hepatocellular carcinoma (HCC)	female immune-deficient BALB/c-nu nude mice	Δ AFAP1-AS1: ↓ tumor weight	(57)
	nude mice	Δ AFAP1-AS1: ↓ tumor weight, ↓ tumor growth, ↓ Ki-67 expression	(58)
Pancreatic cancer (PC)	nude mice	Δ AFAP1-AS1: ↓ tumor volume, ↓ tumor weight	(36)
	male/female BALB/C nude mice	Δ AFAP1-AS1: ↓ tumorigenicity, ↓ EMT process	(63)
	female BALB/c nude mice	CUB-induced Δ AFAP1-AS1: ↓ tumor volume, ↓ tumor weight, ↓ tumor growth	(65)
Pancreatic ductal adenocarcinoma (PDAC)	nude mice	Δ AFAP1-AS1: ↓ tumor volume, ↓ tumor weight	(66)
Renal cell carcinoma (RCC)	female BALB/c athymic nude mice	Δ AFAP1-AS1: ↓ tumor volume, ↓ tumor weight	(67)
Melanoma	male BALB/c nude mice	Δ AFAP1-AS1: ↓ tumor volume, ↓ tumor weight, ↓ tumor size	(71)
Tongue squamous cell carcinoma (TSCC)	female BALB/c athymic nude mice	Δ AFAP1-AS1: ↓ tumor growth, ↓ tumor weight, ↓ tumor size	(73)
Oral squamous cell carcinoma (OSCC)	male BALB/c nude mice	Δ AFAP1-AS1: ↓ tumor volume, ↓ tumor weight	(74)

(Δ: knock down or deletion).

## Clinical Studies

Except from a single low-sample size study in gastric cancer which reported down-regulation of AFAP1-AS1 in tumoral tissues versus nearby samples (6), other studies consistently reported over-expression of AFAP1-AS1 in different neoplastic tissues compared with non-neoplastic tissues of the same origin (**Table 3**). Even in the mentioned study, levels of AFAP1-AS1 were higher in patients who showed lymphatic or vascular invasion in comparison with those without these properties (6). Moreover, different statistical methods have been applied to assess correlations between expression level of AFAP1-AS1 and clinical outcomes, all of them reporting significant impact of up-regulation of this lncRNA on increasing malignant behaviors of tumors and decreasing patients’ survival. In pancreatic cancer, up-regulation of AFAP1-AS1 has been associated with lymph node involvement, perineural invasion, and poor clinical outcome. An in silico analysis of TCGA data of breast cancer patients has revealed AFAP1-AS1, as a differentially expressed lncRNA in basal tumors whose expression levels are associated with poor survival. Expression of this lncRNA has also been associated with hormone receptors status, HER2 expression, and PAM50 classification (81).

**Table 3:** Outlines of studies that appraised levels of AFAP1- AS1 in clinical setting.

Tumor type	Numbers of clinical samples	Expression (Tumor *vs*. Normal)	Kaplan-Meier analysis	Univariate cox regression	Multivariate cox regression	Clinicopathologic characteristics of patients	Reference
Non-small Cell Lung Cancer (NSCLC)	44 NSCLC patient tissues and ANCTs	high	_	_	_	_	(8)
	165 NSCLC patients, 118 benign lung tumor tissues, and 173 healthy samples	high	_	_	_	Paired t test: AFAP1- AS1 was correlated with pathological grade, TNM staging and metastatic ability.	(9)
	GEO analysis	high	_	_	–	_	
	92 pairs of NSCLC tissues and ANCTs	high	Patients with high levels of AFAP1-AS1 had poorer OS.	Histological grade, TNM stage, and AFAP1-AS1 expression were identified as three prognostic factors.	Histological grade, TNM stage, and AFAP1-AS1 expression were independent predictors for OS in NSCLC patients.	Chi-square test: Relative levels of AFAP1-AS1 were associated with tumor burden.	(10)
	7 NSCLC tumor tissues and ANCTs	high	_	_	_	_	(11)
	126 NSCLC patients and 60 healthy controls	high	_	_	_	Mann–Whitney U test: High serum levels of AFAP1-AS1 were strongly associated with DM, LNM, poor clinical stage, and larger tumor size.	(75)
	82 pairs of NSCLC tissue and ANCTs	high	_	_	_	_	(76)
	52 NSCLC patients	high	AFAP1-AS1 down-regulation was correlated with improved survival time.	_	High expression level of ASAP1-S1 was an indicator of poor survival.	_	
Non-small Cell Lung Cancer (NSCLC)	96 pairs of lung cancer tissues and ANCTs	high	AFAP1‐AS1 over-expression was related with short OS and PFS.	_	_	_	(37)
	GEO and TCGA analysis: _	high	_	_	_	_	
	121 NSCLC patients and 79 healthy controls	high	AFAP1‐AS1 over-expression was related with short OS.	_	AFAP1-AS1 was an independent prognostic indicator for NSCLC patients.	Chi-square test: AFAP1-AS1 expression was influenced by clinical stage, smoking history, infiltration extent, LNM and distant metastasis.	(77)
	36 studies: 6267 NSCLC patients	high	_	_	_	_.	(78)
	TCGA analysis: 465 LUAD patients and 49 ANCTs	high	_	_	_	_	(79)
	53 newly diagnosed LUAD tissues and ANCTs	high	_	_	_	_	
	20 pairs of LUAD and LUSC tumor tissues and ANCTs	high	_	_	_	_	(80)
	TCGA analysis: 57 paired LUAD and normal samples and 16 paired LUSC and normal samples	high	_	_	_	_	
Lung cancer	98 pairs of lung cancer tissues and ANCTs	high	_	_	_	Patients with high levels of AFAP1-AS1 had poor histology type, great tumor size, LNM, distant metastasis, and advanced TNM stage.	(38)
	GSE31210 analysis: 226 primary lung cancer samples and 20 normal lung samples	high	High levels of were associated with poor OS.	_	_	_	(12)
	GSE19804 analysis: 60 pairs of lung cancer tissues and ANCTs	high	_	_	_	_	
	GSE27262 analysis: 25 pairs of tumor tissues and ANCTs	high	_	_	_	_	
	GSE18842 analysis: 46 pairs of tumor tissues and ANCTs	high	_	_	_	_	
	GSE37745 analysis: 106 lung cancer biopsies	high	High levels of were associated with poor OS.	_	_	_	
	187 paraffin-embedded lung cancer tissues and 36 normal lung specimens	high	High AFAP1-AS1 expression was tightly correlated with poorer OS.	_	_	_	(16)
Lung cancer	36 lung adenocarcinoma tissue samples and ANCTs	high	High levels of AFAP1-AS1 were associated with shorter DFS.	_	_	_	(39)
Breast cancer (BC)	160 pairs of breast cancer tissues and ANCTs	high	The 3-years OS of patients with high AFAP1-AS1 expression was lower.	AFAP1-AS1 expression, tumor grade, TNM stage, and LNM were Significant factors.	High level of AFAP1-AS1 was correlated with the malignant features.	_	(40)
	20 pairs of breast cancer tissues and ANCTs	high	_	_	_	_	(17)
	TCGA analysis: _	high	_	_	_	_	(18)
	31 pairs of TNBC tissues and ANCTs	high	High levels of AFAP1-AS1 were correlated with poorer DFS and OS.	_	AFAP1-AS1 could be regarded as an independent prognostic factor in TNBC.	_	(20)
	TCGA analysis: _	high	High expression of AFAP1 was correlated with short surviavl in patients with Luminal B, HER2 +, and basal tumors and worse OS Luminal A and HER2 + tumor subtypes.	_	_	_	(81)
	8 pairs of TNBC tissues and ANCTs	high	_	_	_	_	(19)
	64 HER-2 positive patients and 40 HER-2 negative patients	Higher in HER-2 positive than HER-2 negative	_	_	_	_	(21)
	51 pairs of tumor tissues and ANCTs	high	_	_	_	Its expression was low in ki-67 negative tumor tissues.	(82)
Osteosarcoma	8 pairs of Osteosarcoma tissues and ANCTs	high	_	_	_	_	(22)
	45 OS tissues and ANCTs	high	Patients who had high AFAP1-AS1 expression level indicated poor OS rate than those who had low AFAP1-AS1 expression level.	_	_	_	(23)
	49 pairs of OS tissues and ANCTs	high	Patients with higher expression of AFAP1-AS1 showed lower OS and PFS rates.	_	_	_	(24)
Esophageal cancer (EC)	42 ESCC tissues and 35 ANCTs	high	_	_	_	_	(30)
	65 pairs of tissues and ANCTs	high	_	_	_	Chi‐squared test: high level of AFAP1‐AS1 was correlated with tumor size and advanced TNM stage.	(41)
	48 pairs of ESCC tissues and ANCTs	high	_	_	_	_	(83)
	162 pairs of ESCC tissues and ANCTs	high	High levels of AFAP1‐AS1 were strongly associated with shorter PFS.	Tumor depth, LNM, TNM stage, dCRT response, and AFAP1‐AS1 expression were associated with PFS and OS.	Tumor depth, dCRT response, and AFAP1‐AS1 expression were independent prognostic factors for PFS. Moreover, high levels of AFAP1‐AS1 indicated unfavorable OS.	Chi‐squared test: higher expression of AFAP1‐AS1 was strongly correlated with LNM, distant metastasis, advanced clinical stage, and lack of response to dCRT.	
Gastric cancer (GC)	20 pairs of GC tissues and ANCTs	high	_	_	_	_	(27)
	52 pairs of GC tissues and ANCTs	high	_	_	_	_	(43)
	91 pairs of primary gastric cancer tissues and their ANCTs	high	Patients with high levels of AFAP1-AS1 showed poor OS than those with low levels.	_	Lymph node metastasis, TNM stage, and AFAP1-AS1 expression levels were independent prognostic factors for OS time.	X2 test: expression of AFAP1-AS1 was associated with LNM and TNM stage.	(25)
	52 pairs of GC tissues and ANCTs	high	Patients with high expression of AFAP1-AS1 had a significantly poorer OS compared to those with low-expression of AFAP1-AS1.	_	_	_	(28)
	30 tumor tissues and ANCTs	down	_	_	_	Levels of AFAP1-AS1 were higher in patients who showed lymphatic or vascular invasion in comparison with those who did not.	(6)
	66 pairs of GC tissues and ANCTs	high	_	_	Expression of AFAP1-AS1, clinical stage, and tumor differentiation could be regarded as the factors that were independently correlated with OS.	Higher expression level of AFAP1-AS1 was correlated with tumor mass, clinical stage, and tumor differentiation.	(44)
	89 GC patients, 55 benign gastric lesion groups, 73 age-matched healthy volunteers	high	_	_	_	Logistic regression analysis: high level of AFAP1-AS1 was significantly correlated with tumor size, TNM stage and LNM.	(45)
	80 pairs of GC tissues and ANCTs	high	Patients with high levels of AFAP1-AS1 had shorter OS than those with low levels of AFAP1-AS1.	_	_	_	(84)
Prostate cancer	30 PCa tissues and corresponding nearby healthy tissues	high	_	_	_	_	(31)
	38 pairs of prostate cancer tissues and ANCTs	high	Patients with high expression of AFAP1-AS1 had lower OS.	_	_	Chi-Square test: AFAP1-AS1 expression was associated with histological grade and distant metastasis.	(32)
Nasopharyngeal carcinoma (NPC)	10 pairs of freshly frozen samples and ANCTs	high	Patients with high expression of AFAP1-AS1 showed lower OS.	_	_	_	(46)
	100 pairs of paraffin-embedded samples and ANCTs						
	96 paraffin-embedded NPC samples	high	Patients with high expression of AFAP1-AS1 had a poor prognosis, with shorter OS.	_	_	Patients with high expression of AFAP1-AS1 were showed distant metastasis when they relapsed.	(85)
	32 nasopharyngeal carcinoma samples and 13 non tumor nasopharyngeal epithelium tissues	high	_	_	_	High expression of AFAP1-AS1 was highly correlated with clinical TNM stages, neck LNM, and T stages of the patients.	(33)
	101 NPC patients and 101 healthy controls	high	_	_	_	_	(86)
	101 NPC patients and 20 chronic nasopharyngitis patients						
	101 NPC patients and 20 asymptomatic EBV carriers						
	23 NPC samples and 7 non-tumor nasopharyngeal epithelium samples	high	_	_	_	_	(47)
	112 paraffin-embedded NPC and 10 NPE tissue samples	high	High expression of AFAP1-AS1 was correlated with poor OS and poor RFS.	_	_	Expression of AFAP1-AS1 was associated with distant tumor metastasis.	
Endometrial carcinoma (EC)	73 pairs of EC tissues and ANCTs	high	_	_	_	_	(48)
Cholangiocarcinoma (CCA)	20 pairs of CCA tissues and ANCTs	high	_	_	_	_	(49)
	56 pairs of tumor tissues and ANCTs	high	Patients with high expression of AFAP1-AS1showed shorter OS.	_	_	High expression of AFAP1-AS1 had positive association with tumor size, vascular invasion, and advance TNM stage.	(50)
Colorectal cancer (CRC)	68 CRC patients and 60 healthy volunteers	high	_	_	_	Chi-squared test: plasma levels of AFAP1-AS1 were correlated with clinical stage.	(51)
	52 pairs of CRC tissues and ANCTs	high	Patients with up-regulation of AFAP1-AS1 had a significantly poorer prognosis.	AFAP1-AS1 expression, tumor size, TNM stage, and distant metastasis were significantly correlated with OS and DFS.	AFAP1- AS1 expression, TNM stage, and distant metastasis were strongly correlated with OS and DFS.	High levels of AFAP1-AS1 were associated with tumor size, TNM stage and remote metastasis.	(52)
	15 pairs of CRC tissues and ANCTs	high	_	_	_	_	(53)
	TCGA analysis: 50 pairs of clinical colorectal cancer tumors and the peritumoral tissues						
	80 CRC tissues and 10 normal colon tissues	high	Patients who had high AFAP1-AS1 mRNA levels indicated worse prognosis compared with those with low.	_	_	_	(54)
Colon Cancer	GEO analysis: _	high	_	_	_	_	(55)
	TCGA-COAD analysis	high	Patients with high expression of AFAP1-AS1 indicated shorter OS and DFS.	_	_	_	
Hepatocellular carcinoma	17 pairs of tumor tissues and ANCTs	high	_	_	_	_	(56)
	17 pairs of HCC tissues and ANCTs	high	Patients with high levels of AFAP1-AS1 showed a shorter median survival time.	_	AFAP1-AS1 expression could be regarded as an independent prognostic factor for OS in HCC patients.	High levels of AFAP1-AS1 were correlated with pathological staging and lymph-vascular space invasion.	(57)
	156 pairs of HCC tissues and ANCTs	high	Patients with low levels of AFAP1-AS1 showed better OS and DFS.	_	_	High levels of AFAP1-AS1 were correlated with tumor size, vascular invasion, and TNM stage.	(58)
Cervical cancer (CC)	TCGA analysis: _	high	Patients with high expression of AFAP1-AS1 expression had a short OS.	_	_	High levels of AFAP1-AS1 were correlated with TNM stage.	(59)
Laryngeal carcinoma	24 pairs of tumor tissues and ANCTs	high	_	_	_	_	(34)
Thyroid cancer	36 pairs of tumor tissues and ANCTs	high	Patients with high expression of AFAP1-AS1 expression had a short OS	_	AFAP1-AS1 expression might be a positive, independent prognostic factor.	_	(60)
Glioma	52 glioma cases and 5 non-tumor control cases	high	High expression of AFAP1-AS1 predicted worse prognosis in glioma patients.	_	_	Expression of AFAP1-AS1 was closely correlated with glioma grading and KPS scores.	(61)
Ovarian cancer (OC)	65 pairs of OC tissues and ANCTs	high	_	_	_	Upregulation of AFAP1-AS1 was correlated with high FIGO stage and resistance response.	(62)
	39 pairs of OC tissues and ANCTs	high	Patients with low expression of AFAP1-AS1 showed greater survival probability.	_	_	Chi-square analysis: Upregulation of AFAP1-AS1 was correlated with FIGO stage.	(35)
Pancreatic cancer (PC)	75 pairs of PC tissues and ANCTs	high	_	_	_	Upregulation of AFAP1-AS1 was positively associated with TNM stage, LNM, and tumor size.	(36)
	GEO analysis: _	high	_	–	_	_	(64)
	63 pairs of PC tissues and ANCTs	high	Patients with high AFAP1-AS1 expression showed a shorter 5-year OS rate.	_	_	Upregulation of AFAP1-AS1 was positively associated with advanced TNM stage, tumor size and LNM.	
Pancreatic ductal adenocarcinoma (PDAC)	8 cases of PDAC tissues and 4 cases of CP tissues	high	_	_	_	_	(66)
	90 pairs of PDAC tissues and ANCTs	high	Patients with high expression of AFAP1-AS1 showed worse OS and PFS.	_	_	Upregulation of AFAP1-AS1 was positively associated with LNM and perineural invasion.	
Renal cell carcinoma (RCC)	60 ccRCC tissues and 20 ANCTs	high	Patients with high expression of AFAP1-AS1 showed worse OS.	_	_	Upregulation of AFAP1-AS1 was positively associated with LNM and TNM stage.	(67)
Gallbladder cancer (GBC)	40 pairs of GBC tissues and ANCTs	high	Upregulation of AFAP1-AS1 indicated a poor prognosis in gallbladder cancer.	_	_	Upregulation of AFAP1-AS1 was positively associated with tumor size.	(68)
Pituitary adenoma	60 pairs of pituitary adenomas tissues and ANCTs	high	_	_	_	_	(70)
Retinoblastoma	58 freshly frozen retinoblastoma tissue samples and 10 non-cancerous retina samples	high	Patients with high expression of AFAP1-AS1 had shorter OS.	High-expression of AFAP1-AS1 was found to be an unfavorable prognostic factor.	High-expression of AFAP1-AS1 was found to be an independent unfavorable prognostic factor.	Upregulation of AFAP1-AS1 was positively associated with tumor bulk as well as choroidal or optic nerve invasion.	(72)
Tongue squamous cell carcinoma	103 pairs of tumor tissues and ANCTs	high	High AFAP1-AS1 expression was related to poor survival.	_	_	Expression level of AFAP1-AS1 was associated with tumor differentiation, T classification, clinical stage, invasion depth, and relapse.	(73)
Oral squamous cell carcinoma (OSCC)	48 pairs of OSCC tissues and ANCTs	high	Patients with high AFAP1-AS1 expression had a poor OS.	_	_	Expression level of AFAP1-AS1 was associated with an advanced clinical stage and LNM.	(74)

(ANCTs, adjacent non-cancerous tissues; OS, Overall survival; DFS, Disease-free survival; PFS, progression free survival; TNM, tumor‐node‐metastasis; dCRT, definitive chemoradiotherapy; DM, distant metastasis; LNM, lymph node metastasis; TCGA, The Cancer Genome Atlas; GEO, Gene Expression Omnibus; KPS, Karnofsky Performance Status; CP, chronic pancreatitis tissues).

Tissue levels of AFAP1-AS1 could be used as a prognostic biomarker with the areas under ROC curves values of 0.86 and 0.93 for forecasting cancer progression in the periods of 6 and 12 months, respectively (66).

The ability of tissue levels of AFAP1-AS1 or its circulatory levels in differentiation of patients’ samples from control samples has been appraised in different types of cancers (**Table 4**). For instance, Li et al. have shown that over-expression of AFAP1-AS1 in serum samples of patients with NSCLC compared with normal controls can be used to distinguish these two sets of samples with an area under the curve (AUC) of 0.759. Combination of expression levels of this lncRNA with those of cyfra21-1 has increased AUC value to 0.860. Moreover, AFAP1-AS1 over-expression has been more prominent in patients with distant or lymph node metastasis, advanced clinical stage, and greater tumor burden (75). Serum levels of AFAP1-AS1 have also been shown to separate gastric cancer patients from controls with higher AUC value compared with conventional markers, i.e. CEA and CA19-9. Notably, serum levels of AFAP1-AS1 have been shown to be reduced following surgical treatment of patients (45).

**Table 4:** Diagnostic value of AFAP1-AS1 in different cancers.

Tumor Type	Numbers of clinical samples	Distinguish between	Area Under Curve	Sensitivity	Specificity	Accuracy	Reference
Non-small Cell Lung Cancer (NSCLC)	126 NSCLC patients and 60 healthy controls	patients with NSCLC vs. healthy controls	0.759	0.693	0.883	0.759	(75)
Breast cancer	160 pairs of breast cancer tissues and ANCTs	Cancer tissues vs. ANCTs	0.736	74%	69%	_	(40)
Esophageal cancer (EC)	162 pairs of ESCC tissues and ANCTs	Cancer tissues vs. ANCTs	0.802	73.3%	79.4%	_	(83)
Gastric cancer (GC)	30 tumor tissues and ANCTs	Cancer tissues vs. ANCTs	0.67	70%	63.3%	_	(6)
	89 GC patients and 73 healthy controls	patients with GC vs. healthy controls	0.820	76.4%	56.2%	67.3%	(45)
	80 pairs of GC tissues and ANCTs	Cancer tissues vs. ANCTs	0.8802	81.25%	83.75%	_	(84)
Nasopharyngeal carcinoma (NPC)	101 NPC patients and 101 healthy controls	patients with NPC vs. healthy controls	0.665	0.640	0.838	_	(86)
	101 NPC patients and 20 chronic nasopharyngitis patients	patients with NPC vs. chronic nasopharyngitis patients	0.625	0.590	0.822	_	
	101 NPC patients and 20 asymptomatic EBV carriers	patients with NPC vs. asymptomatic EBV carriers	0.620	0.592	0.819	_	

ANCTs, adjacent non-cancerous tissues; ESCC, esophageal squamous cell carcinoma.

## Discussion

AFAP1-AS1 has been found to be up-regulated in almost all kinds of malignant tissues. This lncRNA has multiple effects in the carcinogenesis process, most of them being exerted through AFAP1-independent manners. Most notably, AFAP1-AS1 can sequester a number of tumor suppressor miRNAs, thus releasing the targets of these miRNAs from inhibitory effects of miRNAs. miR-139-5p, miR-545-3p, miR-497-5p, miR-145, miR-2110, miR-4695-5p, miR-26a, miR-498, miR-155-5p, miR-195-5p, miR-512-3p, miR-423-5p, miR-545-3p, miR‐320a, miR-107, miR-384, miR-133a, miR‐146b‐5p, miR-103a-3p and miR-653-5p are among miRNAs which have been found to be sequestered by AFAP1-AS1 through functional studies in different types of cancer cells. Notably, the interaction between AFAP1-AS1 and miR-497 has been verified in breast cancer and osteosarcoma. Moreover, similar interaction has been verified between this lncRNA and miR-145 in breast cancer and oral squamous cell carcinoma.

In fact, AFAP1-AS1 has multiple binding sites for miRNAs, thus regulating expression of a wide array of miRNAs. It is not clear whether binding of this lncRNA with a certain miRNA affects its interactions with other miRNAs. The crosstalk between AFAP1-AS1 and miRNAs can regulate activity of signaling pathways, angiogenic processes as well as EMT.

AFAP1-AS1 can indirectly influence activity of some cancer-related pathways such as EGFR/AKT, Wnt/β-catenin, PTEN/p-AKT, RhoA/Rac2 and PI3K/AKT. The effects of this lncRNA on Wnt/β-catenin, EGF/AKT and PI3K/AKT are mediated through sponging miR-4695-5p, miR-139-5p and miR-103a-3p, respectively. However, its effects on other pathways might be exerted in an independent manner from miRNAs sponging.

Lung cancer, nasopharyngeal carcinoma, colorectal cancer and cholangiocarcinoma are among cancers in which the interaction between AFAP1-AS1 and AFAP1 has been verified. However, the results of these studies are conflicting. For instance, AFAP1-AS1 silencing has been shown to increase expression of AFAP1 in a single study in lung cancer cells (12), while another study in this type of cancer has shown its effect on enhancement of expression of AFAP1 (11). Moreover, in a single study in MCF-7 breast cancer cells, AFAP1-AS1 silencing has not affected AFAP1 levels or actin filament integrity (40). Therefore, future studies are needed to elaborate the mechanistical impacts of AFAP1/AFAP1-AS1 interactions.

AFAP1-AS1 can affect response of cancer cells to a variety of anti-cancer modalities ranging from conventional chemotherapeutics to targeted therapeutics such as trastuzumab. Therefore, measurement of expression levels of this lncRNA can guide clinical oncologists to find the most appropriate therapeutic option for each patient. AFAP1-AS1 can also affect EMT and stemness of cancer cells, thus promoting their metastatic ability and increasing the propensity to tumor recurrence.

From a prognostic point of view, AFAP1-AS1 levels have been associated with tumor depth, tumor differentiation, TNM stage and other determinants of patients’ survival, thus could be used as markers for prediction of clinical outcomes of patients with a variety of malignant conditions. Diagnostic application of AFAP1-AS1 has been appraised in several types of cancers, with the best results being obtained from studies in gastric and esophageal cancers.

Cumulatively, AFAP1-AS1 is a prototype of cancer-related lncRNAs that regulates carcinogenesis not only through modification of expression of its sense transcript, but also through a variety of other methods such as miRNA sequestering and epigenetically affecting expression of tumor suppressor genes.

## Author Contributions

SG-F and BH wrote the draft and revised it. MT designed and supervised the study. TK and MM collected the data and designed the figures and tables. All authors contributed to the article and approved the submitted version.

## Conflict of Interest

The authors declare that the research was conducted in the absence of any commercial or financial relationships that could be construed as a potential conflict of interest.

## Publisher’s Note

All claims expressed in this article are solely those of the authors and do not necessarily represent those of their affiliated organizations, or those of the publisher, the editors and the reviewers. Any product that may be evaluated in this article, or claim that may be made by its manufacturer, is not guaranteed or endorsed by the publisher.
